# Altered Functional Connectivity in a Triple-Network Model in Autism With Co-occurring Attention Deficit Hyperactivity Disorder

**DOI:** 10.3389/fpsyt.2021.736755

**Published:** 2021-12-02

**Authors:** Kai Wang, Ke Li, Xiaoyu Niu

**Affiliations:** ^1^Department of Pediatrics, First Affiliated Hospital, Zhengzhou University, Zhengzhou, China; ^2^Department of Child Healthcare, Children's Hospital Affiliated to Zhengzhou University, Zhengzhou, China

**Keywords:** autism, attention deficit hyperactivity disorder, co-occurring, triple-network model, default mode network

## Abstract

**Purpose:** This study aimed to explore alterations in functional connectivity (FC) within and between default mode network (DMN), central executive network, and salience network in autism spectrum disorder (ASD) with co-occurring attention deficit hyperactivity disorder (ADHD).

**Method:** A total of 135 individuals' date of the Autism Brain Imaging Data Exchange II was used to compare the ASD+ADHD group with the ASD group in relation to the abnormal within-network and between-network connectivity of the ASD group relative to the TD group; consequently, the correlation analysis between abnormal FC and behavior was performed.

**Results:** The ASD+ADHD group exhibited decreased within-network connectivity in the precuneus of the ventral DMN compared with the ASD group. Among the three groups, the ASD+ADHD group showed lower connectivity, whereas the ASD group had higher connectivity than the TD group, although the effect of the separate *post hoc* test was not significant. Meanwhile, the ASD+ADHD group showed increased between-network connectivity between the ventral DMN and dorsal DMN and between the ventral DMN and left executive control network, compared with the ASD and TD groups.

**Conclusion:** Dysfunction of DMN in the “triple-network model” is the core evidence for ASD with co-occurring ADHD.

## Introduction

Autism spectrum disorder (ASD) and attention deficit hyperactivity disorder (ADHD) are defined by symptom-based classification. As described in the Diagnostic and Statistical Manual of Mental Disorders (DSM-5), ASD exhibits abnormal behavioral symptoms of social/communication deficits and restricted and repetitive behaviors, whereas ADHD is defined by attentional and/or hyperactive/impulsive traits (1). The new edition of the DSM-5 allows the diagnosis of comorbid ASD and ADHD in clinical practice (2). Both ASD and ADHD display few clear links between diagnostic criteria and specific neurobiological alterations (3, 4). There are few publications describing consistent phenotypic variations in people with ASD and co-occurring ADHD.

In recent years, the development of functional magnetic resonance imaging (fMRI), a common neuroimaging technique, has provided a promising tool for investigating cognitive dysfunction. Several resting-state fMRI (rs-fMRI) studies have demonstrated that ASD or ADHD is related to atypical patterns of functional connectivity (FC) in large-scale brain networks (5–9). Of the many stable intrinsic brain networks, Menon proposed a triple-network model, which consists of three core neurocognitive networks: the default mode network (DMN), central executive network (CEN), and salience network (SN) as the three most important intrinsic networks for human brain activation. The DMN, considered a task-negative network, includes a collection of brain regions that deactivate reliably during cognitive task performance; its nodes are the medial prefrontal cortex and posterior cingulate cortex (PCC) (10). The CEN, often characterized as a task-positive network, encompasses a frontoparietal system anchored in the dorsolateral prefrontal cortex and posterior parietal cortex, which shows strong co-activation during the performance of cognitively demanding tasks (11). Traditionally, the SN, with key nodes in the dorsal anterior cingulate cortex and frontoinsular cortex, is thought to be involved in the processes of information filtering, detection, and integration (12). The triple-network approach examines “core” brain networks supporting cognitive, perceptual, affective, and social functions and was thought to be abnormally organized in many psychiatric and neurological disorders (13). In recent years, separate research on the human brain functional networks of people with ADHD (14) or ASD (15), the triple-network model has been widely used, and some common brain connectivity issues have been discovered. Specifically, the dysfunction of the DMN plays an important role in the social impairments of people with ASD (16), and the abnormal state switching and cognitive control of people with ADHD are related to the aberrant within-connectivity of the DMN, SN, CEN, and attention networks (6, 17). In addition, ASD and ADHD share the common brain network dysfunction characterized by different coupling patterns of the temporoparietal cortices in the DMN with SN and the dorsal attention network (18). However, the number of current studies related to ASD with co-occurring ADHD—with regard to the FC of the triple-network model—is scarce.

In this study, we applied the triple-network model to investigate the specific alterations of both within-network and between-network FC of the three core neurocognitive networks in the ASD with co-occurring ADHD (ASD+ADHD group) and ASD without co-occurring ADHD (ASD group) using rs-fMRI while identifying the relationship to clinical symptoms. We used independent component analysis (ICA) to identify the brain regions of the DMN, SN, and CEN according to the Stanford Functional Imaging in Neuropsychiatric Disorders (FIND) Lab. We hypothesized that altered connectivity existed both within and between these three core brain networks and predicted that specific FCs would explain the brain-behavior relationship that contributed to explaining the phenotypes of the ASD with ADHD co-occurring, thus demonstrating the diagnosis and potential therapeutic biomarkers of such people.

## Materials and Methods

### Subjects

In our analyses, we included data from four sites of an open-access multi-site image database [Autism Brain Imaging Data Exchange II (ABIDE II), http://fcon_1000.projects.nitrc.org/indi/abide/abide_II.html] with most subjects meeting the following criteria: first, we included individuals with a full-scale IQ and near full-brain coverage structural and rs-fMRI scan data and excluded individuals with excessive head motion characterized by a mean framewise displacement (FD) of more than 0.30 mm; next, we divided the people into two groups (ASD+ADHD and ASD) on the basis of the psychiatric comorbidity information on the ABIDE II website; and finally, from each site, we selected from the included dataset the same number of individuals matching in terms of age- and IQ-matched typical development (TD) with the other two groups. These inclusion criteria and an additional step for matching the three groups at each site resulted in a cohort of 135 individuals (45 ASD+ADHD, 45 ASD, and 45 TD), with whom the three groups were matched by age, sex, and full IQ (FIQ) in the scanner (see Table 1). A selection flowchart for the participants is provided in Supplementary Figure 1.

**Table 1:** Participant demographics.

	**Mean (SD) [Range]**		
	**ASD + ADHD (*N* = 45)**	**ASD (*N* = 45)**	**TD (*N* = 45)**
Age, years	11.2 (4.1) [5.9–26.6]	11.1(4.8) [5.3–34.8]	11.0(2.9) [5.9–23.8]
FIQ	104.0 (16.2) [74–138]	106.0(15.9) [78–136]	106.1(10.3) [85–132]
Sex	Male (*N* = 36) Female (*N* = 9)	Male (*N* = 36) Female (*N* = 9)	Male (*N* = 36) Female (*N* = 9)
SRS_TOTAL_T (N_ASD+ADHD_ = 42; N_ASD_ = 44; N_TD_ = 40)	80.3 (11.2) [54–107]	73.8 (12.2) [42–101]	43.7 (5.1) [34–56]
SRS_AWARENESS_T	74.6 (11.2) [45–97]	68.3 (13.6) [43–100]	43.1 (8.2) [32–65]
SRS_COGNITION_T	74.4 (12.1) [39–99]	69.6 (11.7) [48–90]	43.1 (5.9) [36–59]
SRS_COMMUNICATION_T	79.2 (11.9) [52–99]	72.1 (12.7) [39–97]	44.2 (5.2) [36–58]
SRS_MOTIVATION_T	74.2 (12.5) [51–102]	69.4 (13.2) [47–97]	46.0 (6.4) [37–58]
SRS_ MANNERISMS _T	78.7 (12.6) [53–104]	74.9 (13.8) [40–104]	44.3 (4.6) [40–58]

*SD, standard deviation; ADOS_2_SA, Autism Diagnostic Observation Schedule, Second Edition (ADOS-2); SA, Social Affect; SRS, Social Responsiveness Scale; Participants from the following sites from ABIDE II were included in the final sample of 135 participants: NYU_1, KKI_1, UCD_1, and OHSU_1*.

### Image Acquisition and Preparation

The acquisition parameters and protocol information of the MRI images are provided in Supplementary Table 1. On these rs-fMRI data, we performed preprocessing using the Data Processing Assistant for Resting-State fMRI toolbox (DPARSF version 2.3), implemented in the MATLAB 2014a platform (19). The preprocessing steps included discarding the first five volumes to allow for magnetization equilibration and participant adaptation to the scanning environment, interleaved slice-timing correction, and head motion correction. Participants without excessive head motion, which was defined by a mean FD of <0.3 mm, were included in further analysis. The images were warped to the Montreal Neurological Institute space, then resampled to 3.0-mm isotropic voxels, and finally spatially smoothed with a 5-mm full width half maximum Gaussian kernel to improve the signal-to-noise ratio and reduce inter-subject variability.

### ICA and Identification of Networks-of-Interest

Group ICA was performed on the overall group preprocessed data (135 subjects) using the InfoMax algorithm, as implemented in the Group ICA of fMRI Toolbox (GIFT) version 4.0 (http://www.icatb.sourceforge.net). The aggregate preprocessed data were written and projected into 29 independent components (ICs), the optimal number of their dimensions estimated by the minimum description length criteria tool within the GIFT software (20). Principal components analysis reduced the dimensions of the data (21), followed by an IC estimation that produced spatial maps and time courses (22). Following this, 100 ICA (ICASSO) ensured the stability of the decomposition (23). Next, GICA3 back-constructed a set of mean group components, resulting from the previous steps into a single subject space (24). Finally, to reflect the measures of within-network connectivity, we converted the intensity values of the spatial z-map of each subject to the z value (25).

After all these steps, 29 spatiotemporal components were obtained (Supplementary Figure 2). To select components reflecting each network of interest (the triple-network model), we statistically compared the spatial map of each IC with a group of covered six of 14 major networks consisting of 90 regions of interest (ROIs) [left executive control network (LECN), right executive control network (RECN), anterior_SN, post_SN, dorsal_DMN, and ventral_DMN] described by a previous study (26). We then calculated Pearson's correlation coefficients for each pairwise relationship with Sort Components in GIFT software and retained the ICs with the highest correlation coefficients as networks of interest for our triple-network model (26). This procedure identified nine ICs corresponding, respectively, to the LECN, RECN, anterior_SN, post_SN, dorsal_DMN, and ventral_DMN; the remaining ICs were eliminated from further analysis.

### Within-Network and Between-Network Connectivity

The z-map of each subject-level component was indexed within-network connectivity measurements. Rs-fMRI data were band-pass–filtered (0.008–0.15 Hz) before the functional network connectivity (FNC) analysis (27), and the FNC software (http://mialab.mrn.org/sofware) was used to examine specific temporal correlations in a nonparametric pairwise manner, with a maximal lagged correlation approach, and calculated lags that were correlated to each other. Thus, all nine RSNs were paired with one another among the triple-networks to obtain 36 pairwise combinations, and the coefficients were then transformed into z-scores using Fisher's z-transformation. The transformed z-scores were indexed the between-network connectivity of each network pair.

### Statistical Analysis

Between-group differences in demographic and clinical assessments were appropriately conducted with MANOVA (age, FIQ, ADOS_score, and SRS_score) or the chi-squared test (sex) using Statistical Product and Service Solutions software (version 25.0). The statistical module in DPARSF was used to perform statistical analysis on the spatial z-map (19). In each network, two-sample *t*-tests with age, site, sex, FIQ, and mean FD as covariates were used to analyze the group differences (ASD+ADHD and ASD) of within-network connectivity. The comparisons were limited to the network mask generated by composing the results of the one-sample *t*-test of each group [*q* < 0.01, false discovery rate (FDR) correction] (see Figure 2A). The significance criterion for between-group comparison was *P* < 0.05, correcting by threshold-free cluster enhancement (TFCE) (5,000 permutations) (28). We then used multivariate analysis of variance (MANOVA) to explore differences between FC values extracted from the above-detected clusters between the three groups with sex, age, FIQ, and mean FD as covariates.

To explore the differences in between-network connectivity, the resulting pairwise correlation coefficients were transformed to Fisher's z-values and then extracted into MANOVA while regressing out site, age, sex, FIQ, and mean FD. In addition, we further explored the differences in aberrant between-network connectivity between the ASD+ADHD and ASD groups using a *post hoc* test.

To examine whether there was a relationship between behavioral problems and abnormal within/between-network connections showing group differences, we used multiple regression analyses to estimate the associations after controlling for the site, age, sex, FIQ, and mean FD. The clinical characteristics of ASD were reflected by the z-scores of the Autism Diagnostic Observation Schedule, Second Edition (ADOS-2)_Social Affect (SA) subscale (29), the Social Responsiveness Scale (SRS), and the subscales (30). Our analysis was limited to the availability of ADOS_2_SA data for 95.6% of the ASD (*n* = 86) subjects (Table 1).

## Results

### Clinical Characteristics

As shown in Table 1, the age, sex and FIQ scores in the three groups were well matched. As expected, compared with the ASD and TD groups, the ASD+ADHD group had significantly higher SRS_total_T scores and higher subscale scores.

### Identification of Network of Interests

**Figure 1:**
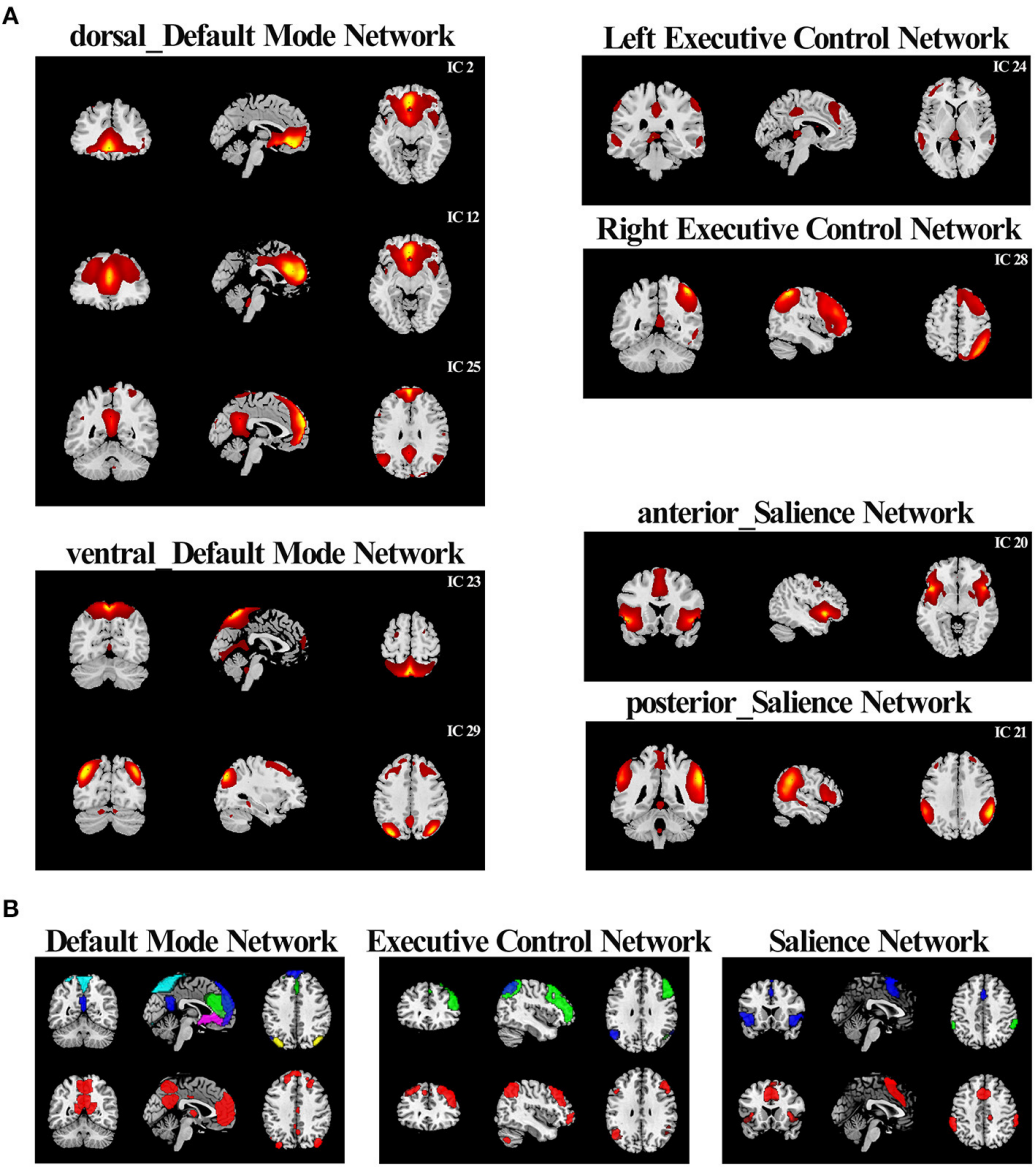
Comparison between the “triple network” of this study and the template networks **(A)** According to the correlation with the triple-network model network templates, the resting-state networks of the current study were divided into six categories. **(B)** The comparison between resting-state networks in current research and the triple-network model templates. RSN combinations of the same category in the current study were drawn in contrasting colors and displayed on the top. The corresponding reference template was drawn in red and displayed on the bottom.

In this study, the nine selected ICs were subsets of the large six major resting-state networks (RSNs) based on 90 ROIs relating to the triple-network model identified by a previous study (31): ICs 2, 12, and 25 were correlated to the dorsal DMN (dDMN); ICs 23 and 29 were correlated to the ventral DMN (vDMN); IC 24 was correlated to the LECN, IC 28 was correlated to the RECN; IC 20 was correlated to ther anterior SN; and IC 21 was correlated to the posterior SN (Figure 1A). We further compared the nine selected ICs in the current study with templates of the triple-network model (Figure 1B).

### Within-Network Dysfunction

**Figure 2:**
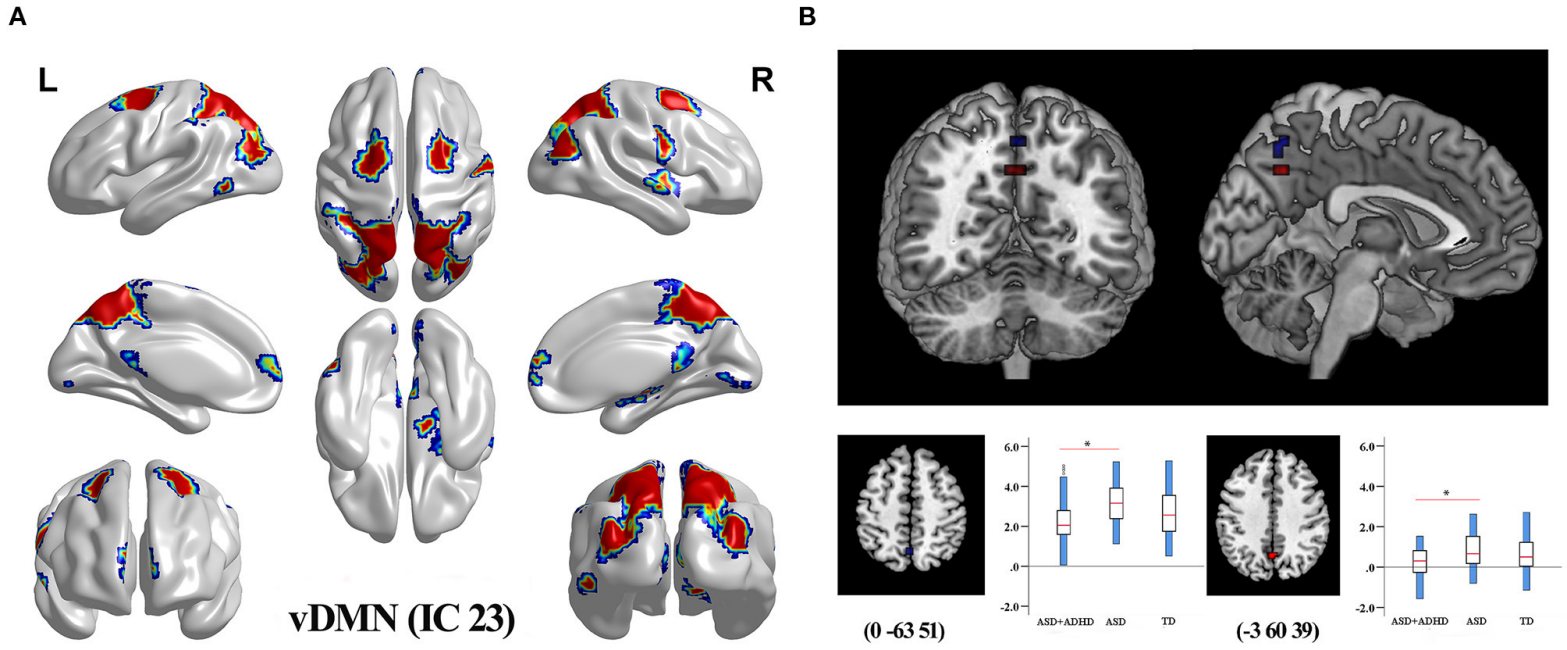
Differences in within-network connectivity in the triple-network model **(A)** The mask of IC 23 belonging to vDMN generated by composing the results of one-sample *t*-test of each group (*q* < 0.01, FDR correction). **(B)** ASD+ADHD group had lower FC in the left and inter-hemispheric precuneus of the vDMN compared with the ASD group.

Group comparisons of the network z-maps showed that, compared with the ASD group (TFCE-corrected), the ASD+ADHD group demonstrated significantly decreased connectivity in the left and inter-hemispheric precuneus of the IC 23, which was identified as the vDMN (Figure 2B, Table 2). There were no significant group differences in the within-network connectivity of the other ICs. Among the three groups, the ASD+ADHD group showed lower connectivity, whereas the ASD group had higher connectivity than the TD group. MANOVA revealed a significant main effect on the group, but the effect of the separate *post hoc* test was not significant (Figure 2B). Therefore, the difference in within-network connectivity observed between the ASD+ADHD group and ASD group is probably due to the sum of two contributions: (1) a decrease in connectivity in the ASD+ADHD group and (2) an increase in connectivity in the ASD group. This increases the delta within-network connectivity between the two groups.

**Table 2:** Brain regions showing significant within-network connectivity differences between ASD+ADHD and ASD groups.

**IC Network**	**Regions (L/R)**	**Peak coordinates (MNI)**			**Voxel number**	**Peak intensity**
		**X**	**Y**	**Z**		
Ventral_DMN(IC 23)	L. Precuneus (BA7)	−3	−60	39	5	−3.6899
	Inter-Hemispheric Precuneus	0	−63	51	5	−3.4222

*IC, independent component; DMN, default mode network; MNI, Montreal Neurological Institute; L, left; R, right; BA, Brodmann area; P < 0.05, TFCE correction with 5,000 permutations*.

### Between-Network Dysfunction

**Figure 3:**
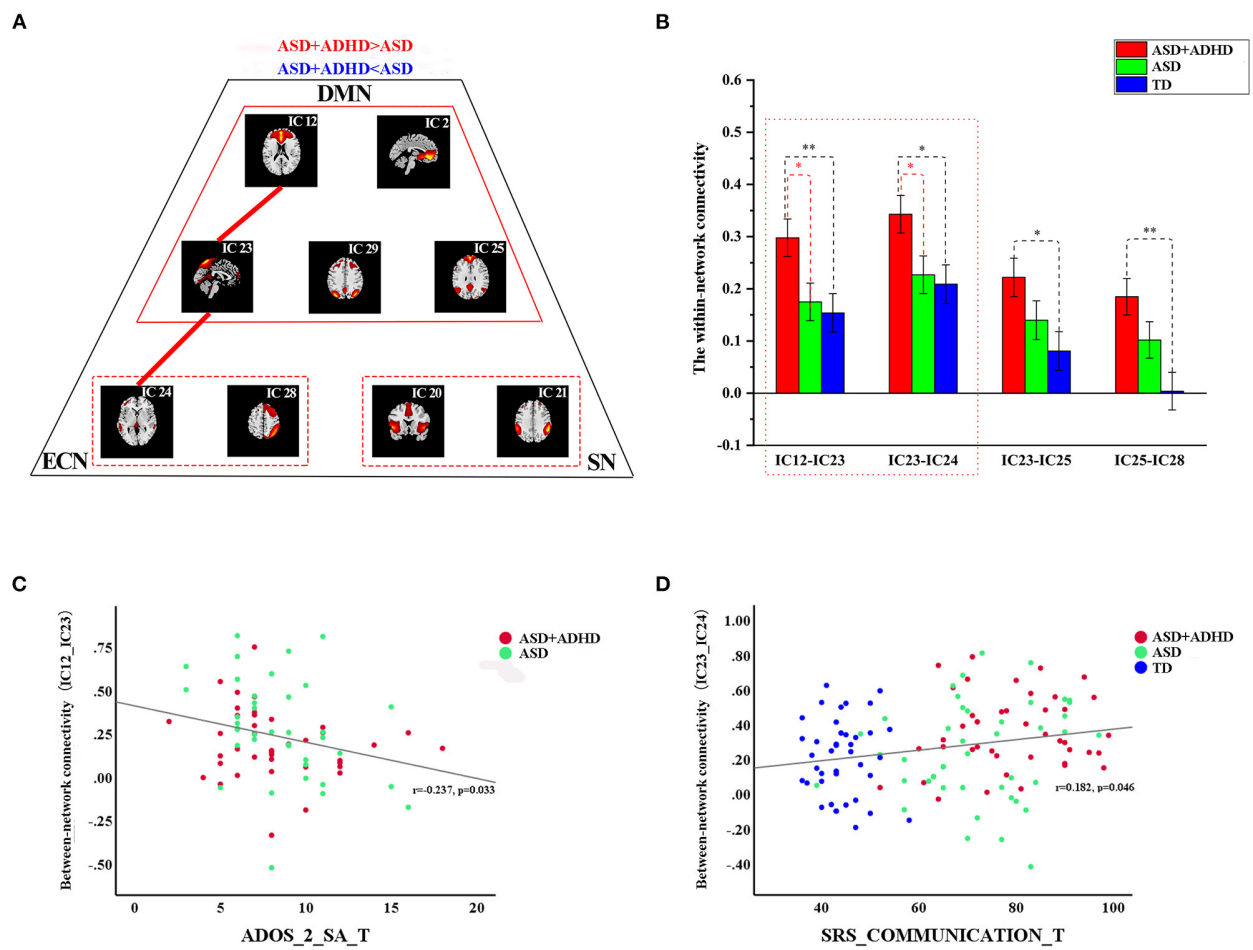
Differences in between-network connectivity and the correlations of clinical measures with abnormal connectivity **(A)** The red line indicates increased FNC strength in ASD+ADHD and the blue line displays the reverse situation. Further details are shown in Table 3. **(B)** Extracted altered between-network connectivity, ASD+ADHD group showed increased dDMN (IC 12)–vDMN (IC 23) connectivity and LECN (IC 24)–vDMN (IC 23) connectivity. **(C)** The altered dDMN (IC 12)–vDMN (IC 23) between-network connectivity by ADOS_2_SA scores. **(D)** The altered dDMN (IC 12)–vDMN (IC 23) between-network connectivity by SRS_communication scores.

The FNC analysis conducted on the between-network connectivity indicated significant differences between the four pairs of correlation coefficients among the three groups (*P* < 0.05; Figure 3B, Table 3). Of the four pairs with altered between-network connectivity, *post hoc* tests showed that ASD+ADHD group had significantly higher dDMN (IC 12)–vDMN (IC 23) and LECN (IC 24)–vDMN (IC 23) between-network connectivity than the ASD and TD groups, which indicated dysfunctional FC between the vDMN and dDMN and between the DMN and ECN; meanwhile, the ASD group had higher connectivity than the TD group, but the effect was not significant (Figure 3A, Table 3).

**Table 3:** Between-network connectivity differences between ASD+ADHD and ASD groups.

**Between-network**	**ASD + ADHD (SE)**	**ASD (SE)**	**TD (SE)**	**F**	***P-*value**	***Post hoc* test**	***p-*value**
**IC 12–IC 23**	0.298 (0.036)	0.175 (0.036)	0.154 (0.037)	4.540	0.012^*^	ASD+ADHD vs. ASD	0.017^*^
						ASD+ADHD vs. TD	0.007^*^
**IC 23–IC 24**	0.343 (0.036)	0.227 (0.036)	0.209 (0.037)	4.008	0.021^*^	ASD+ADHD vs. ASD	0.024^*^
						ASD+ADHD vs. TD	0.012^*^
**IC 23–IC 25**	0.222 (0.037)	0.140 (0.037)	0.081 (0.038)	3.477	0.034^*^	ASD+ADHD vs. TD	0.010^*^
**IC 25–IC 28**	0.185 (0.035)	0.102 (0.035)	0.004 (0.036)	6.370	0.002^*^	ASD+ADHD vs. TD	0.001^*^

*IC, independent component; SE, standard error; TD: typical development*.

* *Significant at P < 0.05*.

### Abnormalities in Network Connectivity Related to Symptoms Severity

As shown in Figures 3C,D, the altered dDMN (IC12)–vDMN (IC23) between-network connectivity was negatively correlated with ADOS_2_SA scores in all people with ASD (*r* = −0.237, *p* = 0.033, *n* = 77), and the altered vDMN (IC23)–LECN (IC24) between-network connectivity was positively correlated with SRS_communication T-scores in all samples (*r* = 0.182, *p* = 0.046, *n* = 126). However, the results were not corrected for multiple comparisons. There was no significant correlation between the abnormal within-network connectivity of the vDMN, and clinical characteristics measured by SRS and ADOS_2_SA scores were not detected.

## Discussion

In this study, we demonstrated the dysconnectivity within and between the SN, DMN, and ECN in ASD with and without co-occurring ADHD, on the basis of the triple-network model. ASD+ADHD group showed decreased within-network connectivity in the left and inter-hemispheric precuneus of the vDMN compared with ASD group. Notably, ASD+ADHD group also demonstrated increased between-network connectivity between the dDMN and vDMN and between the vDMN and LCEN, which suggested the presence of atypical dynamic interactions of the triple network in the ASD with co-occurring ADHD. These results provide evidence of aberrant connectivity patterns across the core neurocognitive networks of triple-network models in ASD with co-occurring ADHD.

Social functioning is a major and central domain of impairment in both disorders, which significantly affects prognosis. Studies have reported that social awareness, social cognition, emotion recognition, and social communication may be poorer in children with ASD+ADHD than those with ASD alone (32). In this study, ASD+ADHD group showed more serious social deficits than those with ASD (Table 1). The networks of interest in the current study were informed by the triple-network hypothesis (13), according to which many psychiatric and neurological conditions were characterized by disorder-specific patterns of increased or reduced function and connectivity in the SN, DMN, and ECNs. The overall pattern observed in our study, showing an underconnectivity within the DMN accompanied by complex patterns of over connectivity between DMN and ECN and between vDMN and dDMN, was relevant to this hypothesis in several respects. As a major functional brain system, the DMN has been proven to play an important role in several psychiatric disorders including ASD (16) and ADHD (33). Previous studies indicate that the precuneus plays a core role not only in the DMN but also more broadly through its engagement under a variety of processing states (34–36). Its role in the DMN has been of particular interest because it shows the highest resting metabolic rate within the network, requiring ~35% more glucose than any other region in the human brain (37). Abnormalities of the precuneus in people with ASD (38) and ADHD (33, 39) have been revealed in numerous studies, one of which suggested that the right calcarine, left superior frontal gyrus (SFG), and DMN nodes (including the precuneus) play important roles in children with ASD and co-occurring anxiety and ADHD (40). The fMRI meta-analyses of cognitive control including 60 fMRI datasets revealed ASD-differentiating medial prefrontal under activation but overactivation in the bilateral ventrolateral prefrontal cortices and precuneus (41); our study also found that the ASD group showed increased within-network connectivity in the precuneus compared with the TD group. A previous study showed that there was diffused hypoconnectivity within the default network in people with ADHD (42), and the lack of overactivation in the PCC/precuneus in people with ADHD during cognitive control was apparent in previous meta-analyses (43) and could potentially be related to psychostimulant exposure, which has been shown to normalize DMN functioning (44). Compared with the TD group, comorbid ADHD may cause hypoconnectivity within the DMN in people with ASD, which increases the delta within-network connectivity between ASD+ADHD and ASD groups. In addition, the ASD+ADHD group showed increased between-network connectivity between the vDMN and dDMN.

New achievements have been made relative to the triple-network aberrant connectivity in other psychiatric conditions, such as schizophrenia. In 2020, Luo et al. (45) found an aberrant task-evoked increase in the influence of the right anterior insula (rAI) on the middle frontal gyrus (MFG) and precuneus. Control signals from the rAI (salience signaling) were abnormally elevated and directed toward both task-positive (ECN) and task-negative brain (DMN) regions, when task-related demands arise in schizophrenia, contributing to working memory deficiency. In our study, we mainly determined the aberrant interaction between the DMN and ECN, but whether it was caused by abnormal salience signals from specific brain regions is still unclear. Further study is needed to identify if there is an abnormality in sensory-salience circuity in the process system in children with ADHD and ASD symptoms. There has also been updated research on dynamic FNC (dFNC) in triple networks of psychiatric conditions. In 2019, Wang et al. (46) found that people with bipolar disorder and major depressive disorder spent more time in sparse connections with decreased dFNC variability between the posterior DMN and the right CEN. It revealed more common but less specific dFNC alterations in both conditions: whether it was the same in ADHD with and without ASD needs to be clarified, which may help us understand their abnormal cognitive functions clinically (46). Supervised convex non-negative matrix factorization was utilized to distinguish different psychiatric conditions in a latent low-dimensional space of the triple brain network, with high classification accuracy on the basis of the extracted structural and functional abnormalities, which inspired our further study (47).

The DMN participates in evaluative social processes (16), mentalizing and theory of mind (48, 49), which is important for the social understanding of others (50). Rather than reflecting the DMN as a unitary, homogenous system, recent imaging studies have tended to show DMN dissociation, with the anterior DMN being more engaged in self-referential and emotional processes and with the posterior DMN being involved in episodic memory and perceptual processing (51, 52). Previous reports have pointed out that disrupted intrinsic DMN organization in children and adults with ASD is related to social deficits (53–55), which is consistent with our study and further suggests that comorbid ADHD may aggravate hyperconnectivity between the dDMN and vDMN and lead to the aggravation of social dysfunction in people with ASD.

As in the proposed triple-network model, the SN, DMN, and ECN did not function independently. The DMN represented the task-negative processing mode for the human brain, whereas the ECN characterized the somewhat contrary task-positive processing mode. Increasingly, research has revealed the important role of cooperation between the SN, ECN, and DMN in maintaining cognitive functions (56, 57). In this study, the levels of DMN-ECN interaction in the ASD+ADHD group were higher than those in the ASD group. In addition, a previous study showed that people with ASD displayed increased connectivity between the DMN and ECN, and the strength of FC decreased with age (58). Overconnectivity in the ASD group observed for DMN–ECN pairings thus partly reflected reduced anticorrelations (rather than robust positive correlations), indicating a reduction in the typical segregation between networks, consistent with some previous reports (59, 60). Notably, the dysfunction of the DMN in the triple-network may be a neurobiological feature of ASD with ADHD co-occurrence and a potential image trait marker to help identify such subgroups of people with ASD. The SN is involved in monitoring behaviorally relevant salient stimuli and interrupting ongoing activity when appropriate, playing a dynamic switching role between the DMN and the ECN, which support self-related (or internally directed) and goal-oriented (or externally directed) cognition, respectively, to guide appropriate responses to salient stimuli (61). So far, we have not found statistically significant differences between the ASD+ADHD and ASD groups.

This study has some limitations. First, our analyses were performed on people with ASD who had comorbid ADHD and people with ASD who did not have comorbid ASD based on the dataset; however, we did not have ADHD symptom scores for the participants. Further research including both ASD and ADHD symptom scores is required. Moreover, this study ignored the diagnoses of other psychiatric comorbidities.

## Conclusions

In conclusion, our data show that, in addition to impaired FC within the DMN in people with ASD and comorbid ADHD, they also demonstrate aberrant functional interactions between the vDMN and dDMN and between the vDMN and LCEN. This triple-network model provides a new and powerful framework for understanding the dysfunctional brain architecture of ASD with co-occurring ADHD.

## Data Availability Statement

The original contributions presented in the study are included in the article/Supplementary Material, further inquiries can be directed to the corresponding authors.

## Ethics Statement

Ethical review and approval was not required for the study on human participants in accordance with the local legislation and institutional requirements. Written informed consent to participate in this study was provided by the participants' legal guardian/next of kin.

## Author Contributions

KW designed this study. KW and KL performed data preprocessing and analysis. XN and KL drafted the manuscript. All authors approved the final version for publication.

## Conflict of Interest

The authors declare that the research was conducted in the absence of any commercial or financial relationships that could be construed as a potential conflict of interest.

## Publisher's Note

All claims expressed in this article are solely those of the authors and do not necessarily represent those of their affiliated organizations, or those of the publisher, the editors and the reviewers. Any product that may be evaluated in this article, or claim that may be made by its manufacturer, is not guaranteed or endorsed by the publisher.
