# Supplementary material for: Altered Functional Connectivity in a Triple-Network Model in Autism With Co-occurring Attention Deficit Hyperactivity Disorder
Source: Front Psychiatry. 2021 Dec 2;12:736755. doi: 10.3389/fpsyt.2021.736755 (PMC8674431; doi:10.3389/fpsyt.2021.736755)
Supplement: Supplementary file 1 [file Table_1.DOCX]

Supplementary Material

# Supplementary Figures and Tables

For more information on Supplementary Material and for details on the different file types accepted, please see [here](http://home.frontiersin.org/about/author-guidelines#SupplementaryMaterial). Figures, tables, and images will be published under a Creative Commons CC-BY licence and permission must be obtained for use of copyrighted material from other sources (including re-published/adapted/modified/partial figures and images from the internet). It is the responsibility of the authors to acquire the licenses, to follow any citation instructions requested by third-party rights holders, and cover any supplementary charges.

## Supplementary Figures

**
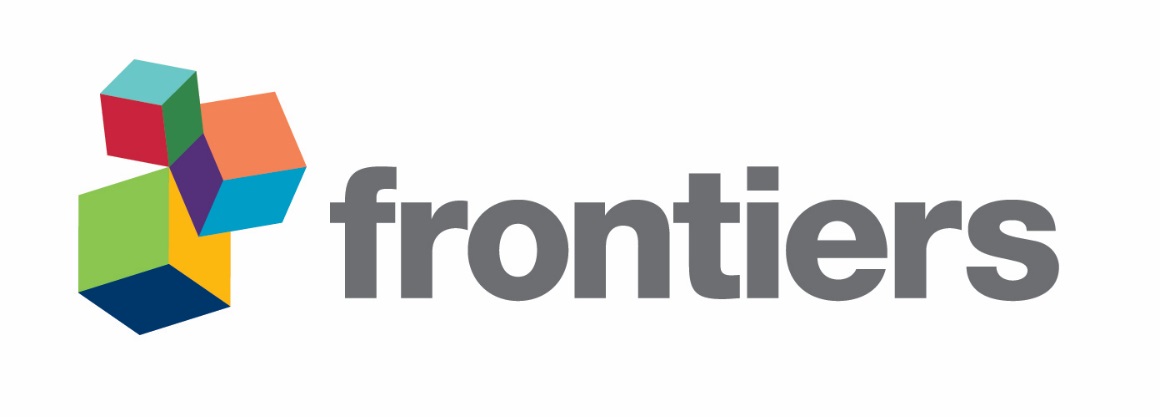
**

**Supplementary Figure 1.** Data were selected from the ABIDE Ⅱ database. Specifically, we selected the participants for this analysis from the sites that featured all the basic inclusion criteria, described in the top two steps of the flowchart: NYU_1, KKI_1, UCD_1, OHSU_1. Additional information about the final sample is reported in Table 1 in the main text.

**Supplementary Figure 2.** 29 rs-fMRI networks extracted by the independent component analysis.

## Supplementary Tables

**Supplementary Table 1 .** The scan parameters of 4 sites from ABIDE Ⅱ

| **Site** | | **NYU_1** | **KKI_1** | **UCD_1** | **OHSU_1** |
| --- | --- | --- | --- | --- | --- |
| **Site location** | | New York, USA | Baltimore, Maryland, USA | Davis, USA | Oregon, USA |
| **The scan parameters** | **Eyes status** | open | open | open | open |
|  | **Number of Measurements (n)** | 180 | 156 | 151 | 120 |
|  | **TR (ms)** | 2000 | 2500 | 2000 | 2500 |
|  | **TE (ms)** | 30 | 30 | 24 | 30 |
|  | **Number of Slices (n)** | 34 | 47 | 36 | 36 |
|  | **Slice thickness (mm)** | 3 | 3 | 4 | 3.8 |

Abbreviations: NYU_1: NYU Langone Medical Center: Sample 1; KKI: Kennedy Krieger Institute; UCD: University of California Davis; OHSU: Oregon Health and Science University; TR: Repetition Time; TE: Echo Time
