# Supplementary figures and images for: Altered Functional Connectivity in a Triple-Network Model in Autism With Co-occurring Attention Deficit Hyperactivity Disorder
Source: Front Psychiatry. 2021 Dec 2;12:736755. doi: 10.3389/fpsyt.2021.736755 (PMC8674431; doi:10.3389/fpsyt.2021.736755)

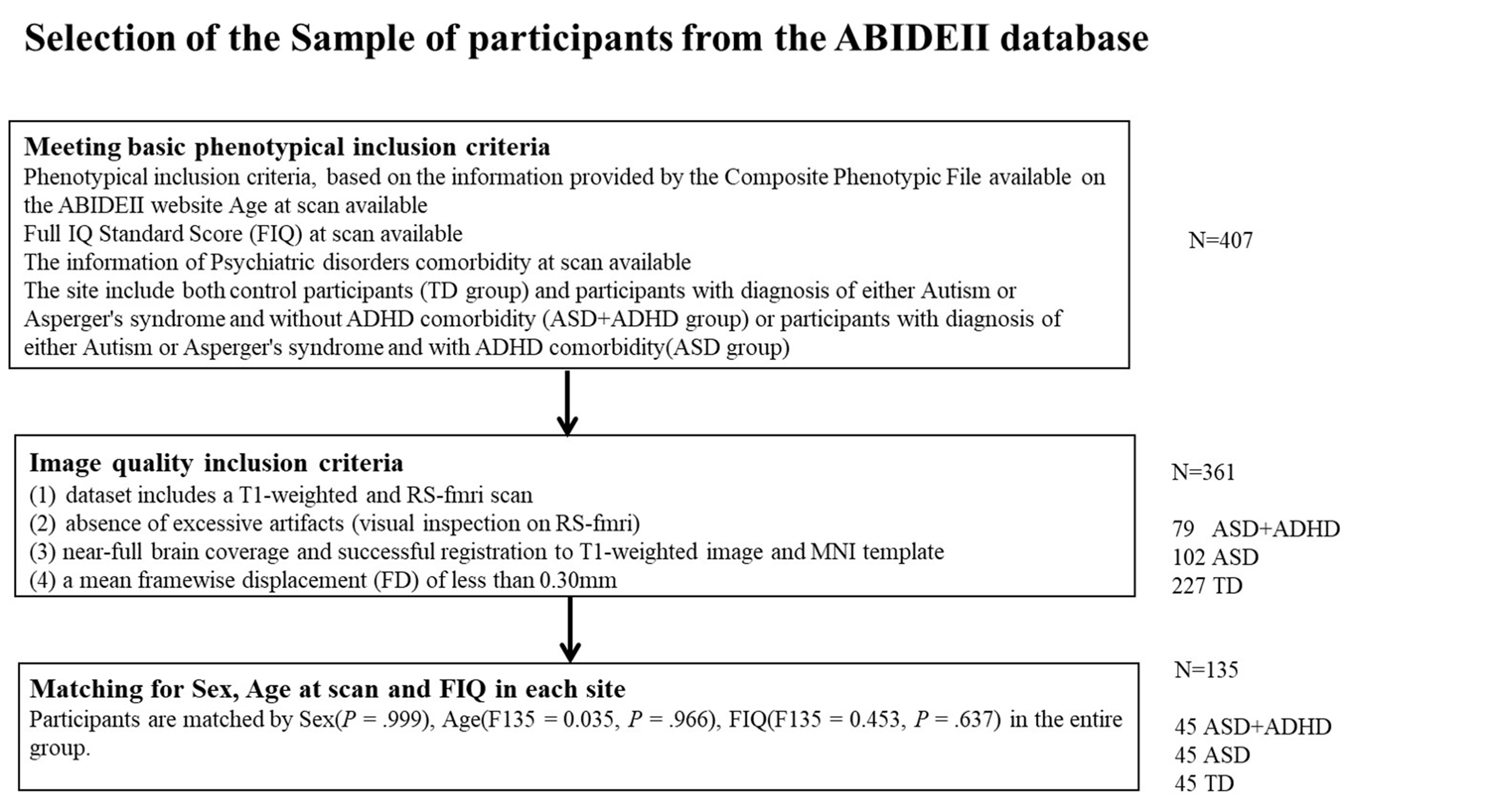

Supplement: Supplementary file 2 [file Image_1.JPEG]

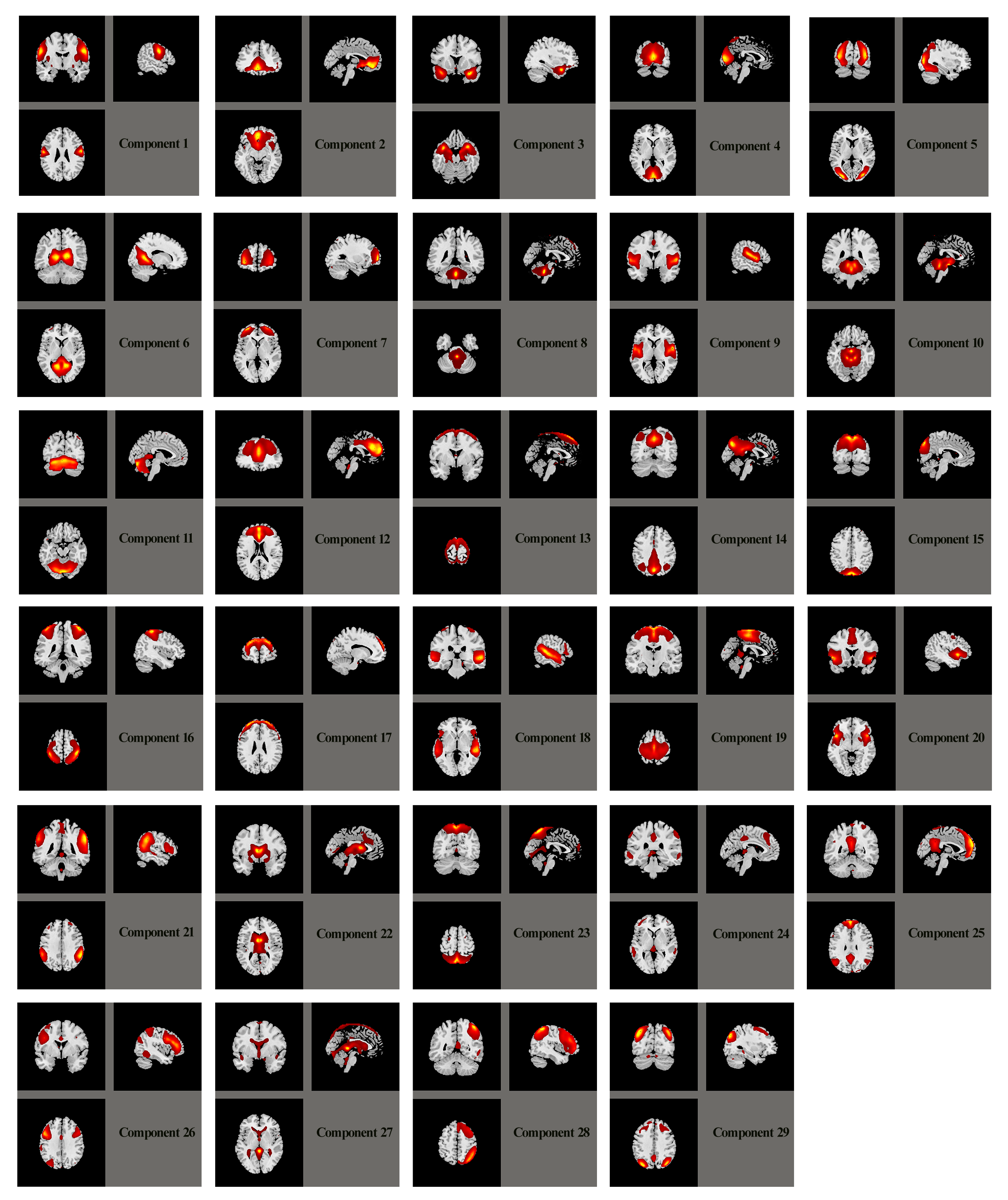

Supplement: Supplementary file 3 [file Image_2.TIF]
